# Liposome encapsulated perfluorohexane enhances radiotherapy in mice without additional oxygen supply

**DOI:** 10.1186/s12967-016-1033-3

**Published:** 2016-09-20

**Authors:** Linfeng Xu, Xuefeng Qiu, Yanting Zhang, Kai Cao, Xiaozhi Zhao, Jinhui Wu, Yiqiao Hu, Hongqian Guo

**Affiliations:** 1 Department of Uroloågy, Affiliated Drum Tower Hospital, School of Medicine, Nanjing University, Nanjing, 210008 China; 2 State Key Laboratory of Pharmaceutical Biotechnology, School of Medicine, Nanjing University, Nanjing, 210093 China; 3 Institute of Urology, Nanjing University, Nanjing, 210008 China

**Keywords:** Perfluorocarbon, Perfluorohexane, Nanoparticles, Radiosensitization, Accumulation, Oxygen supply

## Abstract

**Background:**

To investigate the effect of perfluorochemical preparations in enhancing radiotherapy, perfluocarbon nanoparticles were by encapsulating perfluorohexane into liposome [lip(PFH)].

**Methods:**

After intravenous injection, lip(PFH) could accumulate in the tumor site over time, with a prominent accumulation in tumor 24 h post injection. X-ray was delivered to the tumor site 24 h after the injection of lip(PFH) under room air. The experimental mice were randomized into four groups: control (saline), lip(PFH) (lip(PFH) only), X-ray (X-ray only), and lip(PFH) + X-ray (lip(PFH) with X-ray radiation). Tumor volume and histology were monitored to assess treatment efficacy.

**Results:**

Tumor growth was significantly reduced in mice received lip(PFH) and X-ray compared with X-ray only. The histological data also revealed more destruction of tumor tissue in lip(PFH) + X-ray group compared with X-ray only. In addition, lip(PFH) did not show any significant tissue damage to major organs or induce significant liver/kidney dysfunction.

**Conclusions:**

Lip(PFH) could accumulate in the tumor site and enhance radiotherapy without additional oxygen supply.

## Background

Radiotherapy is one of the most frequently used anti-cancer therapies. Radiotherapy applies high-energy ionizing radiation to destruct tumor cells by producing a free radical on the DNA to induce DNA damage. Usually, this free radical enters into a competition for oxidation or reduction. Oxidation primarily induced by oxygen could fix the damage while reduction primarily induced by—SH-containing compounds could restore the DNA damage [1]. Therefore, the presence of oxygen is crucial for the efficacy of radiotherapy. However, hypoxia has been well identified in most animal tumor models, as well as human tumors, leading to radioresistance in most solid tumors [1–3]. It also has been demonstrated that the radioresistance of solid tumors is positively correlated with oxygen levels in tumor reflected by partial pressure of oxygen [4], as well as by the expression of endogenous markers of hypoxia, such as hypoxia-inducible factor 1α (HIF-1α) [5, 6].

Since the presence of tumor hypoxia is the key mechanism involved in radioresistance of solid tumors, several strategies have been attempted to sensitize hypoxic cells to radiotherapy by supplying oxygen to solid tumor, including high oxygen-content gas breathing under hyperbaric conditions [2], modified hemoglobin [7, 8], and perfluorochemicals [9–13]. Due to their high oxygen capacity, perfluorocarbons (PFCs) were developed into perfluorochemicals emulsions to supply oxygen to tumors and thus enhance radiotherapy in preclinical studies [9–13]. However, the combination with carbogen breathing (95 % O_2_: 5 % CO_2_) before and during the radiation was indispensable, suggesting very limited efficacy of these perfluorochemical emulsions to supply oxygen [9, 14]. These perfluorochemicals emulsions were intended to increase the oxygen content in circulation since they were originally designed as a blood substitute [9] or a ultrasound contrast agent [11]. In fact, tumor hypoxia is mainly due to the disturbed microcirculation and deteriorated diffusion, so that oxygen in circulating perfluorocarbons could not enter hypoxic tissue successfully [1].

**Fig. 1:**
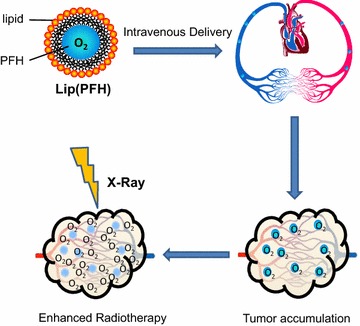
Schematic of the nano-oxygen-carrier for the enhanced radiotherapy

In our previous study, PFC nanoparticles were designed to enhance photodynamic therapy (PDT) [15]. Interestingly, improved efficacy of PDT was achieved without additional oxygen apply before or during the NIR irradiation. In the present study, PFC nanoparticles were developed to supply oxygen to the tumor site and therefore enhance radiotherapy. Perfluorohexane (PFH) was encapsulated into liposome to develop nanoparticles. Radiation was delivered at the time point when lipsome(PFH)[lip(PFH)] accumulates in the tumor site prominently. Compared with the previously reported perfluorochemical emulsions for sensitization of radiotherapy, the efficacy of lip(PFH) is significantly improved since it delivery oxygen directly to the tumor site (Fig. 1). To the best of our knowledge, this is the first perfluorochemical preparation which could enhance radiotherapy without additional oxygen supply.

## Methods

### Chemicals and reagents

Lecithin and cholesterol were purchased from Aladin Industrial Corporation, and DSPE-PEG2000 was obtained from A.V.T. Pharm. Ltd. (Shanghai, China). PFH was purchased from Bailingwei Tech Co., Ltd. (Beijing, China). IR780 was obtained from Sigma-Aldrich Chemical Corporation. All animals used in the present study were purchased from Medical Center of Yangzhou University (Yangzhou, Jiangsu, China).

### Preparation and characterization of lip(PFH)

Lip(PFH) was prepared by ultrasonication according to our previously reported study [15]. Briefly, liposome colloidal suspensions were prepared by dissolving 3.79 mg DSPE-PEG2000 (4.28 mg cholesterol and 24.65 mg lecithin in dichloromethane. The organic solvent was removed through rotary evaporation to form a thin lipids film on the glass vial. Thereafter, the lipid film was hydrated with 1.4 ml pure water by ultrasonication (XO-650D, China) for 10 min in ice bath. After that, 0.6 ml PFH were gradually appended under ultrasonication at 325 w/min for 3 min in ice bath to form 2 ml lip(PFH) (30 v/v % PFH). For in vivo tracking, IR780, a near infrared (NIR) dye, was added in dichloromethane to form lip(PFH + IR780) [15].

The morphology of lip(PFH) was characterized by transmission electron microscopy (TEM, H-7650, Hitachi, Japan). Particle size distribution of liposomes was detected by Nanoparticle Size Analyzer (ZEN3600, Malvern). B ultrasound was used to further confirm that PFH was encapsulated into liposome.

### Animals and tumor model

All experiments were approved by the Institutional Review Board of Nanjing University. Male Balb/c mice (~25 g) were used to establish allograft tumor model according to protocols described before [16]. Briefly, 1 × 10^7^ CT26 cells (mice colon cancer cells) in 0.2 ml PBS were subcutaneously injected into right flank of mice. Fourteen days later, the tumor was isolated and cut into small blocks (~1 mm^3^). One tumor block was implanted into the right flank of each healthy mouse to establish tumor model. When the size of tumor was around 80 mm^3^, the tumor bearing mice were used for each experiment.

### Tumor accumulation and imaging

To investigate the distribution of lip(PFH) in tumor bearing Balb/c mice, B ultrasound was used to detect the signal of PFH in vivo. Lip(PFH) (1.5 v/v %, 8 ml/kg) was intravenously injected into tumor bearing mice. The B ultrasound image of tumor was taken from 6 to 72 h after injection.

To further confirm the accumulation of lip(PFH) in tumor site, IR780, a NIR dye, was loaded into lip(PFH) [lip(PFH + IR780)] for in vivo tracking. IVIS Lumina imaging system (Xenogen Co., USA, excitation/emission, 745 nm/ICG) was used to detect the NIR fluorescent signal in vivo. Lip(PFH + IR780) (1.5 v/v % PFH, 8 ml/kg) was intravenously injected into tumor bearing balb/c mice via tail vein. The whole body optical imaging was taken 24 h after intravenous injection. Exposure time was set to 1 s. Anesthetic animals were placed on an animal plate and heated to 37 °C. Images were analyzed using IVIS Living Imaging Software. To further confirm the distribution of lip(PFH + IR780), three mice were sacrificed 24 h post injection and different organs including heart, liver, spleen, lung, kidney, and tumor were isolated for NIR fluorescent signal detection.

### Radiation therapy

The tumor bearing mice were randomly assigned to receive saline (8 ml/kg) (saline + X-ray) or lip(PFH) (1.5 v/v PFH %, 8 ml/kg) (lip(PFH) + X-ray) 24 h before radiotherapy. For radiation delivery, mice were anesthetized using 2 % pentobarbital sodium, and received local radiation to the tumors at a dose rate of 1.25 Gy/min using 6 MV X-rays by linear accelerator (Clinac 600C, Varian Medical System, Palo Alto, CA, USA) under room air. Additional two groups of mice received intravenous injection of saline (saline) or lip(PFH) [lip(PFH)] without X-ray radiation.

### Tumor growth time (TGT) assay

After the treatment, the tumor size of each mouse was recorded every day using calipers. Tumor volume (V) was calculated as V = d2*D/2 where D and d are the longest and shortest diameter of the tumor respectively. Tumor response to the treatment was evaluated by calculating TGT, defined as the time required for the initial tumor volume to grow tenfold after treatment [9]. Tumor growth delay (TGD) time was calculated by the following formula: TGD of each tumor = TGT (treated) of each tumor-average TGT of control group.

### Histological analysis

Twenty-four hours after treatment, three mice in each group were sacrificed and tumors were harvested, fixed in 10 % formalin, processed routinely into paraffin, sectioned (5 μm) for histological analysis. Tissue sections were stained with hematoxylin and eosin (H&E) using a commercial available kit (Jiancheng Biotechnology, Nanjing, China) according to the protocol provided by the manufacturer.

Quantitative determination of apoptosis in tumor sections was assessed by a terminal transferase-mediated dUTP nick-end labeling (TUNEL) assay using an In Situ Cell Death Detection Kit (Roche, Basel, Switzerland). After dewaxing and rehydration, the tissue sections were permeabilized with 0.1 % Triton X-100 for 10 min. Incubation with label solution was used to detect the apoptotic cells according to the instructions. Apoptotic score was achieved by counting the number of positive nuclei in 10 random fields.

### Toxicity of lip(PFH) to CT26 cells and healthy mice

The toxicity of lip(PFH) was investigated using CT26 cells. Cell-viability was determined by cell-counting kit-8 (CCK-8) assay (Jiancheng Biotechnology, Nanjing, China). Briefly, CT26 cells were plated in 96-well flat-bottomed plates with a concentration of 4000 cells per well and allowed to grow overnight prior to incubation with lip(PFH) with different concentrations from 1.25 v/v PFH % to 5 % v/v PFH %. 24 h later, culture medium containing lip(PFH) was changed with fresh RPMI-1640 medium and CCK-8 was added to each well for an additional 2 h. The cell viability was determined by measuring the absorption at 450 nm using a microplate reader (Safire, TECAN, USA).

The toxicity of lip(PFH) to healthy male mice was conducted by injecting lip(PFH) (1.5 v/v PFH %, 8 ml/kg) via tail vein. Mice injected with saline were set as control. Two weeks after injection, major organs from each mouse were harvested, fixed in 10 % formalin, processed routinely into paraffin, sectioned, stained with hematoxylin and eosin (H&E) and examined by a digital microscope. Examined tissues include heart, liver, spleen, lung and kidney. In addition, blood serum was collected for biochemistry assay of liver and renal function using commercially available kits.

### Statistical analysis

Data were analyzed using Prism 4 (GraphPad Software, San Diego, CA, USA) and expressed as mean ± standard deviation. Multiple groups were compared using multivariate analysis of variance or one-way ANOVA followed by the Tukey–Kramer test for post hoc comparisons. Student t test was used to compare the difference between two groups. Statistical significance was set at P < 0.05.

## Results

### Preparation and characterization of lip(PFH)

**Fig. 2:**
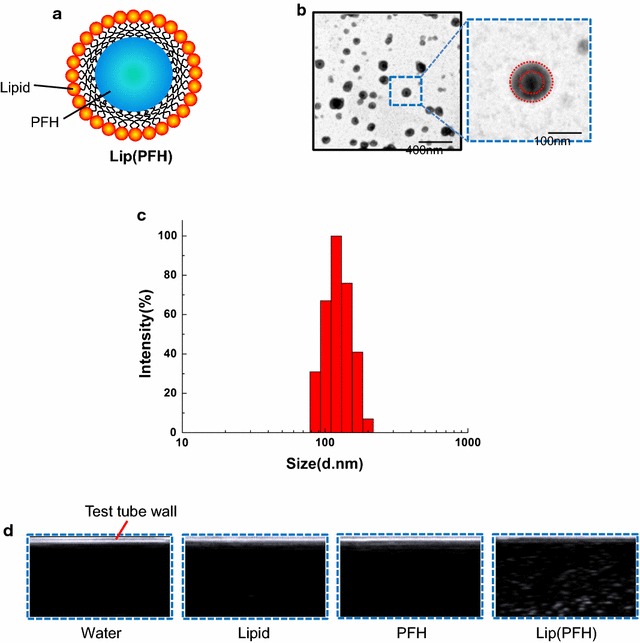
Characterization of the nano-oxygen-carrier. **a** Schematic of the nano-oxygen-carrier. **b** TEM image of the nano-oxygen carrier. **c** Size distribution of the nano-oxygen-carrier. **d** Ultrasound images of the nano-oxygen carrier and other groups tested in 5 ml plastic test tubes

PFH was encapsulated into liposome to form lip(PFH) with a core of PFH and a shell of lipid (Fig. 2a). TEM showed that these oxygen-carriers were spherical vesicles (Fig. 2b) with an average diameter of 100 nm. From the results of dynamic light scattering (DLS), the size of lip(PFH) was 106.1 nm with low variation (Fig. 2c), which was consistent with that measured by TEM. As shown in Fig. 2d, enhanced contrast signal was observed in lip(PFH) by using B ultrasound, further confirming that PFH was encapsulated into liposome.

### Accumulation of lip(PFH) in tumor site

**Fig. 3:**
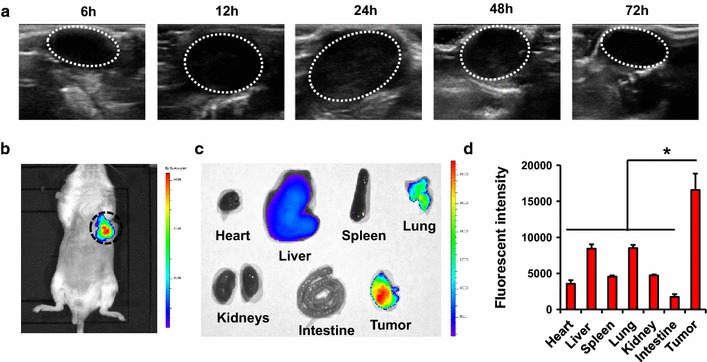
Accumulation of the nano-oxygen-carrier in tumor site. **a** Ultrasound imaging of the nano-oxygen carrier in tumor-bearing mice after intravenous injection of lip(PFH). Images were taken at 6–72 h post-injection. Tumors were circled by *white dash line*. **b** Near-infrared imaging of IR780 in tumor-bearing mice after intravenous injection of lip(IR780 + PFH). Imagines were taken 24 h post-injection. Tumors were circled by *blue dash line*. **c** Near-infrared imaging of IR780 in the different organs from tumor-bearing mice 24 h post-injection of lip(IR780 + PFH). **d** Quantitation of near-infrared signals in the organs from tumor-bearing mice 24 h post-injection of lip(IR780 + PFH). *P < 0.05 compared with tumor

Since lip(PFH) could be detected by B ultrasound, the accumulation of lip(PFH) in tumor site was confirmed by B ultrasound. As shown in Fig. 3a, the ultrasound imaging showed that lip(PFH) tended to accumulate in the tumor over time, with prominent accumulation in tumor 24 h post injection.

Lip(PFH + IR780) was prepared to further confirm the accumulation of PFH in the tumor site. As shown in Fig. 3b, the accumulation of lip(PFH + IR780) in tumor site was detected 24 h post injection, which was consistent with the results obtained from B ultrasound. To further confirm the accumulation of lip(PFH + IR780) in tumor, fluorescence in tumor and main organs (Fig. 3c, d) was detected 24 h after injection of lip(PFH + IR780). The results revealed that the tumors had stronger NIR fluorescence signal, confirming the accumulation of lip(PFH) in tumor.

### The effect of lip(PFH) on sensitizing tumor to radiotherapy

**Fig. 4:**
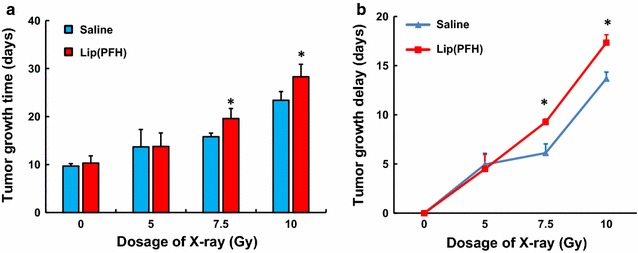
The effect of the nano-oxygen-carrier in delaying the growth of tumor. **a** Tumor growth time of CT26 tumor. Growth time of CT26 tumor produced by different dosage (0, 5, 7.5, 10 Gy) of X-ray radiation 24 h after administration of saline or lip(PFH). Each *column* represents mean ± SD of 6 mice. *P < 0.05 compared with saline group. **b** Growth delay time of CT26 tumor. Tumor growth delay of CT26 tumor produced by by different dosage (0, 5, 7.5, 10 Gy) of X-ray radiation 24 h after administration of saline or lip(PFH). Each *column* represents mean ± SD of 6 mice. *P < 0.05 compared with saline group

TGT and TGD were used to evaluate the tumor response to the treatment. According to the results from multivariate analysis of variance, the TGT was affected by X-ray (P < 0.01), Lip(PFH) (P < 0.01),and X-ray*Lip(PFH) (P < 0.05) significantly. Furthermore,compared with that in mice treated with saline or lip(PFH) only, TGT and TGD in mice treated with X-ray was significantly increased. In addition, significantly increased TGT, and TGD were observed when lip(PFH) was injected 24 h before X-ray radiation, suggesting significantly enhanced tumor response to X-ray (Fig. 4a, b).

**Fig. 5:**
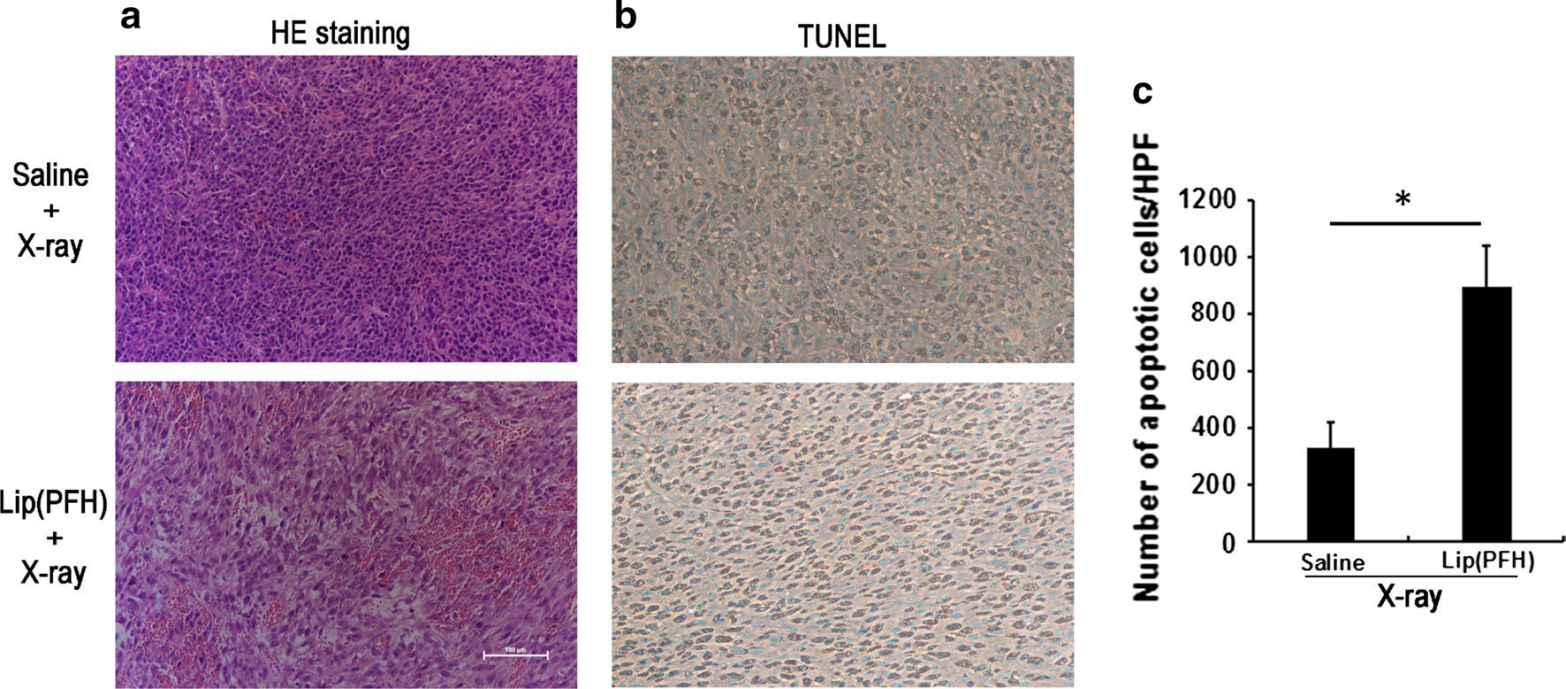
The effect of the nano-oxygen-carrier on the histology of tumor. **a** H&E staining of the tumor section. Twenty four hours after IV injection of saline or lip(PFH), the tumor was irradiated with X-ray (10 Gy). One day after irradiation, tumors were dissected for H&E staining. The *white bar* indicates 200 μm. **b** TUNEL staining of the tumor section. Twenty four hours after IV injection of saline or lip(PFH), the tumor was irradiated with X-ray (10 Gy). One day after irradiation, tumors were dissected for TUNEL staining. *Brown signal* indicates apoptotic cells. **c** Quantitation of apoptotic cells in saline and lip(PFH) groups. *P < 0.05 compared with saline group

To further investigate the tumor response to the treatment, 3 mice in each group were sacrificed 48 h after injection and the tumor from saline + was harvested for histological analysis. Compared with the saline + X-ray group, more necrosis was observed in the tumor section from lip(PFH) + X-ray group (Fig. 5a). As shown in Fig. 5b, c, tumors from mice injected with lip(PFH) 24 h before radiation contained a significantly greater number of TUNEL-positive apoptotic cells compared with that from mice treated with saline and X-ray. This data further confirm that administration of lip(PFH) before X-ray irradiation enhance tumor response to X-ray.

### Toxicity of lip(PFH) to CT26 cells and healthy mice

**Fig. 6:**
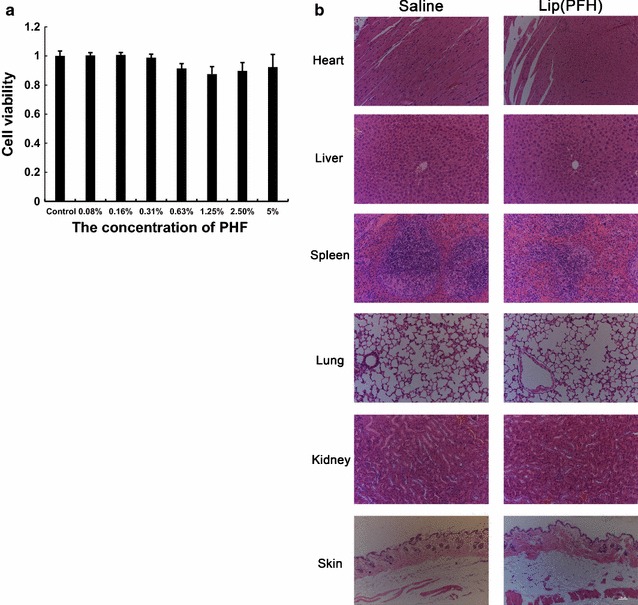
Toxicity of the nano-oxygen-carrier on healthy mice. **a** Cell viability of CT26 cells treated by different concentration of lip(PFH). Cell viability was indicated by CCK-8. **b** H&E staining images of major organs including heart, liver, spleen, lung, kidney and skine. Two weeks after IV injection of saline or lip(PFH), the mice were sacrificed and the tumors were dissected for H&E staining. The *white bar* indicates 200 μm

The potential toxicity of lip(PFH) to CT26 cells was evaluated by CCK-8 assay. From the results, no dark toxicity of the micelles was observed in CT26 cells after 24 h incubation with lip(PFH) even at the highest concentration (5 % v/v PFH %) (Fig. 6a).

The potential toxicity of nano-oxygen-carrier was conducted by injecting lip(PFH) (1.5 v/v PFH %, 8 ml/kg) to healthy Balb/c mice via tail vein. The histological data revealed that there was no noticeable tissue damage in all the major organs of mice at therapeutic dose (1.5 v/v PFH %, 8 ml/kg) in comparison with control group (Fig. 6b). The results suggest no obvious toxicity in histological level of mice is induced by lip(PFH).

The main liver function markers including alanine aminotransferase (ALT), aspartate aminotransferase (AST), alkaline phosphatase (ALP), gamma-glutamyl transpeptidase (GGT), adenosine deaminase (ADA), total bile acid (TBA), total bilirubin (TBIL), albumin/globin ratio (A/G), lactate dehydrogenase (LDH) (Fig. 6b) as well as kidney function markers including serum urea (UREA), serum creatinine (CREA) and uric acid (URIC) (Fig. 6c) were all measured. All these results obtained at 2 weeks post injection did not show significant difference compared with control group. These results suggested no obvious hepatic or kidney disorder of mice induced by lip(PFH) (Tables 1, 2).

**Table 1:** Serum level of liver function markers in mice 2w after injection of saline or lip(PFH) (1.5 v/v PFH %, 8 ml/kg)

	ALT (U/l)	AST (U/l)	AKP (U/l)	GGT (U/l)	ADA (U/l)	TBA (μmol/l)	TBIL (μmol/l)	A/G	LDH (U/l)
Saline	48.86 ± 3.79	86.16 ± 13.25	176.8 ± 16.32	1.0 ± 0.15	4.2 ± 0.38	0.8 ± 0.07	0.5 ± 0.06	1.22 ± 0.09	361 ± 38.1
Lip(PFH)	49.67 ± 6.89	87.68 ± 6.89	169.6 ± 15.74	1.0 ± 0.09	4.2 ± 0.47	0.8 ± 0.06	0.5 ± 0.08	1.23 ± 0.12	324 ± 46.5

n = 3

**Table 2:** Serum level of renal function markers in mice 2w after injection of saline or lip(PFH) (1.5 v/v PFH %, 8 ml/kg)

	UREA (mmol/l)	CREA (μmol/l)	URIC (μmol/l)
Control	8.86 ± 1.02	4.13 ± 1.02	13.87 ± 1.53
Lip(PFH)	8.12 ± 0.94	3.98 ± 0.89	13.56 ± 1.04

n = 3

## Discussion

Due to its ability of carrying oxygen, perfluorochemical emulsions were originally designed as blood substitutes to replace the O_2_ carrying capacity of the blood to provide adequate O_2_ perfusion, and thus to resuscitate patients in hemorrhagic shock after traumatic injuries [17]. Since hypoxia is the key mechanism involved in the redioresistance of solid tumors, PFCs were also developed for radiosensitization since 1980s.Fluosol-DA has been shown to be effective in enhancing the response of several solid tumors to radiotherapy both in preclinical studies [9, 18–22] and some clinical studies [17, 23]. Due to the poor stability of Fluosol-DA, several other improved perfluorochemical emulsions have been developed for radiosensitization [11–13]. Usually, PFCs were delivered shortly before radiation (e.g. 1 h) and intended to increase the oxygen content in circulation and thus enhance radiotherapy in the most reported studies [9, 11, 14, 24, 25]. Despite the effect of perfluorochemical emulsions on radiosensitization, the efficacy of perfluorochemical emulsions seemed to be very limited since oxygen breathing (95 % O_2_ or 100 % O_2_) before and during the radiotherapy was indispensable. As blood substitutes, perfluorochemical emulsions were originally designed to replace the O_2_ carrying capacity of the blood to provide adequate O_2_ perfusion, and thus to resuscitate patients in hemorrhagic shock after traumatic injuries [17]. Therefore, PFCs were delivered shortly before radiation (e.g. 1 h) and intended to increase the oxygen content in circulation and thus enhance radiotherapy in the most reported studies [9, 11, 14, 24, 25].

However, the vascular network is pathological in most solid tumors [26]. Different from that in normal tissues, vascular network in tumor is chaotic, dilated, and tortuous [3, 27]. Due to the accelerated proliferation of cancer cells, hypoxic regions arise in tumor tissue distant from blood vessels. In addition to these regions of chronic hypoxia (also defined as diffusion-limited hypoxia), temporary closure of blood vessels can develop acute hypoxia (perfusion-limited hypoxia) [1, 9, 28]. Since the diffusion distance of oxygen is typically around 150 μm [2], very limited oxygen in circulating PFCs could diffuse to the tumor tissue which is distant from the vessels. In addition, temporary obstruction of tumor vessels occurs, resulting failure of delivering oxygen in circulating PFCs to tumor tissue. That might be the reason why the efficacy of perfulorochemical emulsions to enhance radiotherapy in the previous studies was very limited, since the oxygen in circulating perfulorochemical emulsions could not diffuse to the tumor site successfully.

In our previously published study, improved PDT was achieved by delivering both oxygen-carrying PFH and photosensitizer to the tumor site [15]. This give us inspiration to enhance radiotherapy by delivering oxygen-carrying PFH into the tumor site. Infrared laser was delivery 24 h after injection of PFH when the PFH arrived prominent accumulation in tumor. This give us inspiration to enhance the efficacy of PFCs for radiotherapy. In the present study, PFC nanoparticles were developed by encapsulating PFH into liposome to supply oxygen directly to the tumor site. X-ray was delivery 24 h after lip(PFH) injection, when lip(PFH) arrived the prominent accumulation in the tumor site. The results indicated that lip(PFH) sensitize the tumor to radiotherapy significantly (Fig. 4) even x-ray radiation was delivered without any additional oxygen breathing. The difference between the saline group and the lip(PFH) group is not that notable but significant. It might be due that only once lip(PFH) was administrated during the present study. Further studies will be conducted to investigate how to enhance this radiosensitization effect of lip(PFH).

The accumulation of lip(PFH) in tumor site is essential for direct supply of oxygen. Data obtained from in vivo NIR fluorescent imaging and B ultrasound shows great accumulation of lip(PFH) in the solid tumor 24 h after intravenous injection (Fig. 3), which was consistent with the results in our previously reported study [15]. This might be mainly due to the nano-size of lip(PFH) which was developed to be with an average diameter of 100 nm. The size of lip(PFH) is small enough to avoid clearance by the reticuloendothelial system (RES) and to permeate into tumor tissue through enhanced permeability and retention (EPR) effect. In addition to the nano-size, the adoption of PEG as a stealth moiety on the surface of lip(PFH) could extend the resident time in vivo [29, 30], further contributing to the great tumor accumulation. Some other mechanisms such as hypoxic conditions in the tumor site recruit lip(PFH) might exit, which need further investigations. After accumulating in tumor site, oxygen in PFH could be released directly to the tumor site, shortening the diffusion distance between oxygen carriers and the tumor cells. In addition, x-ray radiation was delivered after accumulation of the oxygen carriers in the tumor site. Therefore, oxygen in PFH could be released directly to the tumor site even the tumor vessels are temporarily obstructed before or during the radiotherapy.

Oxygen release in the tumor site is another essential factor for the enhance radiosensitization. From both in vivo NIR fluorescent imaging and B ultrasound data, PFC nanoparticles were observed to be accumulated in tumor site with a prominent accumulation in tumor 24 h post injection. Therefore, x-ray radiation was delivered 24 h post injection of lip(PFH) in the present study. Unlike hemoglobin, where oxygen is bound to the molecule to form chelates, oxygen is physically dissolved in PFCs without any chemical process [31]. Therefore, oxygen solubility expressed as partial pressure (pO_2_) approaches a linear function in PFCs, unlike the saturation level in hemoglobin. In addition, the gas molecules situate themselves in the spaces between the molecules [31]. The uptake and release of oxygen from PFCs are completely reversible, and the rate is twice as fast as chelation to hemoglobin. After accumulating in the tumor site, the dissolved oxygen could be released because of the low pO_2_ in tumor tissue. Because of its high capacity of oxygen, PFH in the tumor site could also collect oxygen from the near tissue when the contained oxygen was exhausted.

## Conclusions

PFC nanoparticles were designed to supply oxygen directly to the tumor site for the enhancement of radiotherapy. Radiation was delivered when the nanoparticles accumulated in tumor site. Improved radiotherapy was achieved without additional oxygen breathing, suggesting new perspectives of applying PFC to enhance radiotherapy.

## Abbreviations

HIF-1α: hypoxia-inducible factor 1α
PFH: perfluorohexane
PFC: perfluorocarbon
PDT: photodynamic therapy
Lip(PFH): lipsome(PFH)
TGT: tumor growth time
TGD: tumor growth delay
H&E: hematoxylin and eosin
CCK-8: cell-counting kit-8
ALT: alanine aminotransferase
AST: aspartate aminotransferase
ALP: alkaline phosphatase
GGT: gamma-glutamyl transpeptidase
ADA: adenosine deaminase
TBA: total bile acid
TBIL: total bilirubin
A/G: albumin/globin ratio
LDH: lactate dehydrogenase
UREA: serum urea
CREA: creatinine
URIC: uric acid
RES: reticuloendothelial system
EPR: enhanced permeability and retention
